# Heatwaves during low tide are critical for the physiological performance of intertidal macroalgae under global warming scenarios

**DOI:** 10.1038/s41598-020-78526-5

**Published:** 2020-12-08

**Authors:** Marta Román, Salvador Román, Elsa Vázquez, Jesús Troncoso, Celia Olabarria

**Affiliations:** 1 Departamento de Ecoloxía E Bioloxía Animal. Facultade de Ciencias Do Mar, Universidade de Vigo, Campus Lagoas-Marcosende, s/n, 36310 Vigo, Pontevedra, Spain; 2 CIM. Grupo de Ecoloxía Costeira, Edificio CC Experimentais, Universidade de Vigo, Campus de Vigo, As Lagoas, Marcosende, 36310 Vigo, Spain

**Keywords:** Ecophysiology, Marine biology

## Abstract

The abundance and distribution of intertidal canopy-forming macroalgae are threatened by the increase in sea surface temperature and in the frequency and intensity of heatwaves caused by global warming. This study evaluated the physiological response of predominant intertidal macroalgae in the NW Iberian Peninsula (*Bifurcaria bifurcata, Cystoseira tamariscifolia* and *Codium tomentosum*) to increased seawater temperature during immersion and increased air temperatures during consecutive emersion cycles. We combined field mensuration and laboratory experiments in which we measured mortality, growth, maximum quantum yield and C:N content of the macroalgae. Air temperature was a critical factor in determining physiological responses and survivorship of all species, whereas high seawater temperature had sublethal effects. *Cystoseira tamariscifolia* suffered the greatest decreases in F_v_/F_m_, growth and the highest mortality under higher air temperatures, whereas *C. tomentosum* was the most resistant and resilient species. Two consecutive cycles of emersion under atmospheric heatwaves caused cumulative stress in all three macroalgae, affecting the physiological performance and increasing the mortality. The potential expansion of the warm-temperate species *B. bifurcata*, *C. tamariscifolia* and *C. tomentosum* in the NW Iberian Peninsula in response to increasing seawater temperature may be affected by the impact of increased air temperature, especially in a region where the incidence of atmospheric heatwaves is expected to increase.

## Introduction

Sublittoral and intertidal canopy-forming macroalgae form the basis of coastal trophic webs and support economic activities such as fisheries and tourism in many coastal regions^1,2^. In the present context of global warming, macroalgae are potentially important contributors to carbon sequestration^3^, although they are also threatened by the expected increases in seawater temperature^4^ and air temperature^5,6^. Gaining an understanding of the vulnerability of macroalgae to global warming is therefore of great ecological and socio-economic concern.

The most likely situation under a baseline scenario for future climate change (i.e. a scenario in which no efforts have been made to reduce greenhouse gas emissions) will be a balance between representative concentration pathways (RCP) 6.0 and 8.5^7^. Under this scenario, it has been estimated that the global temperature is likely to increase by between 2.25 and 3.7 °C by 2081–2100, relative to the 1986–2005 values. Moreover, climate change will probably lead to increased frequency, intensity and duration of heatwaves^7–9^.

Temperature strongly influences the survival, recruitment, growth and reproduction of seaweeds^10^. Previous studies have examined how the physiology of intertidal fucoids and kelps is affected by increases in seawater temperature^11,12^ or increases in air temperature^13–15^. The effect of acute thermal stress events on macroalgae is of high importance, as physiological traits of intertidal macroalgae, such as growth, nutrient uptake and photosynthetic performance, are negatively affected by marine heatwaves^16–19^. Direct exposure of the fronds to intense sunlight, increased air temperature due to atmospheric heatwaves and desiccation during emersion at low tide are also detrimental to the physiological performance of macroalgae^6,20,21^, decreasing photosynthetic activity and leading to oxidative stress^22,23^. In temperate zones, aerial thermal stress generally peaks in summer, on clear calm days when low tide occurs in mid-afternoon^24^. If such conditions persist during few consecutive days, physiological performance of macroalgae may be negatively affected as the conditions will lead to harsh thermal and desiccation stresses^15,20^. Thus, for example, the kelp *Laminaria digitata* (Hudson) J. V. Lamouroux showed reduced resilience after repeated exposure to atmospheric heatwaves on emersion^25,26^. These two types of warming are, therefore, important drivers in the global distribution of intertidal canopy-forming macroalgae, leading to species decline and range shifts^27–30^ with further consequences on the structure and functioning of communities and entire ecosystems^16,31–33^. This is the case of the fucoid *Fucus serratus* Linnaeus, which has undergone a westward shift in distribution in northern Spain in the last few decades, so that it is now almost entirely limited to scattered populations^34^ as a consequence of the increased seawater and air temperatures^20,35^. Such changes have altered the structure of benthic invertebrate assemblages and shortened the length of the food chain^36^.

The combination of atmospheric and marine heatwaves has been reported to cause high mortality and abundance losses of *Durvillaea* spp. along the New Zealand coast^37^. However, there are few laboratory experiments that have studied the combined effects of both stressors (see^20^). Physical stressors may act additively, synergistically or antagonistically^38^. Synergistic interactions between increased air and seawater temperatures may have greater impacts and unexpected negative responses as the response will be greater than that predicted from the effect of each individual stressor^38^. In the fucoid *F. serratus*, air and seawater temperatures act additively^20^, which suggests that these stressors may exert some general effects on macroalgae. Consecutive periods of exposure at low tide within a single spring tidal cycle can also affect the physiological response of some species^21,25,26^. The effect of different temperatures during consecutive emersion cycles is yet unknown and it should therefore be taken into account by replicating low tide exposure experienced in the field^25^. Further research is required to address the cumulative effects of thermal stress on the physiology of intertidal macroalgae, the additive effects of stressors and the potential consequences for the future distribution of these organisms.

In the NW Iberian Peninsula, the air temperature has increased by 0.5 °C·decade^−1^ since 1974 and the coastal surface seawater temperature has increased by 0.15 °C·decade^−1^ since 1985^39^. These changes are expected to intensify in the future^40,41^; for example, the seawater temperature in the Iberian upwelling is expected to increase by 2.4 °C in the period 2070–2100^41^. Distributional shifts due to increasing seawater temperature have already occurred along the northern Iberian Peninsula, with a clear northward expansion of warm-water species over the last decade^30,42^, together with a decline in some habitat-forming species such as kelps and fucoids^27,43,44^. Increased seawater temperature has led to changes in the distribution of populations of the intertidal fucoids (Ochrophyta) *Bifurcaria bifurcata* R. Ross and *Cystoseira tamariscifolia* Hudson and of the green macroalga *Codium tomentosum* Stackhouse. Populations of these species currently co-occur, covering large areas of the rocky intertidal shore on emergent rock or in rock pools along the northwestern Iberian Peninsula^45,46^. The abundance and distribution of both *B. bifurcata* and *C. tamariscifolia* have increased in a westward direction on the northern coast of Spain, and the observed patterns have been linked to recent increases in sea surface temperature^28,44,47^. Predictive models for global warming scenarios indicate that *B. bifurcata* will continue to expand throughout the whole of the NW Iberian coast^30^, whereas the distribution of *Codium* species such as *Codium fragile* subsp. *fragile* (Suringar) Hariot in the NW Atlantic may remain unchanged^29^. Nevertheless, the physiological effects of increasing temperatures on the cosmopolitan species *C. tomentosum* are unknown.

The aim of this study was to determine the physiological responses of three intertidal macroalgae that are predominant on NW Iberian rocky shores to marine and atmospheric heatwaves during consecutive emersion cycles simulating two spring tidal cycles. We combined field measurements with a novel laboratory experiment in which we recreated the conditions experienced by macroalgae during typical summer consecutive spring tides, where individuals may be exposed to repeated cycles of thermal stress during low tide and to different seawater temperatures. The study evaluated various physiological responses, including mortality rate, growth, photosynthetic performance (as F_v_/F_m_) and C:N content of *B. bifurcata, C. tamariscifolia* and *C. tomentosum* to increased air and seawater temperatures of the magnitude expected under global warming projections. These variables were chosen as suitable proxies for stress because F_v_/F_m_ indicates short-term regulatory responses, whereas growth integrates the intermediate term physiological effects, and the C:N ratio indicates the intermediate term effects on nutrient assimilation and use^48^. We expected to observe lower F_v_/F_m_^18^ and growth^49^, higher C:N ratios^50^, higher probability of mortality^37^, differences between species due to their different eco-physiological traits^51^ and also additive effects of stressors, in response to higher air and seawater temperatures. We also expected cumulative stress to act as a key driver determining different responses of macroalgae to physiological stress.

## Results

### Field measurements

#### Environmental conditions

The average seawater temperature recorded by a data logger deployed at the study site in July and August was 17.02 ± 1.05 °C. The day of the field measurements the average seawater temperature was 18.18 ± 0.61 °C, the average amount of light measured on bare rock was 2956 ± 516 μmol photon m^−2^ s^−1^ and the air temperature reached a maximum of 38 °C (Supplementary Fig. S1). The mean temperature below the canopies was 25.68 ± 0.12 °C for *B. bifurcata*, 25.76 ± 0.16 °C for *C. tamariscifolia* and 25.79 ± 0.24 °C for *C. tomentosum.* Humidity (%) below the canopies was 86.13 ± 0.21 for *B. bifurcata*, 86.41 ± 0.29 for *C. tamariscifolia* and 88.27 ± 0.31 for *C. tomentosum.* Temperature below the canopies did not differ between the macroalgae (GLM for temperature: χ^2^ = 0.15, df = 2, *p* = 0.928, n = 3), whereas humidity was significantly higher below *C. tomentosum* (GLM for humidity: χ^2^ = 37.49, df = 2, *p* < 0.001, n = 3) (Supplementary Fig. S2).

#### *∆Weight and ∆F*_*v*_*/F*_*m*_

**Figure 1:**
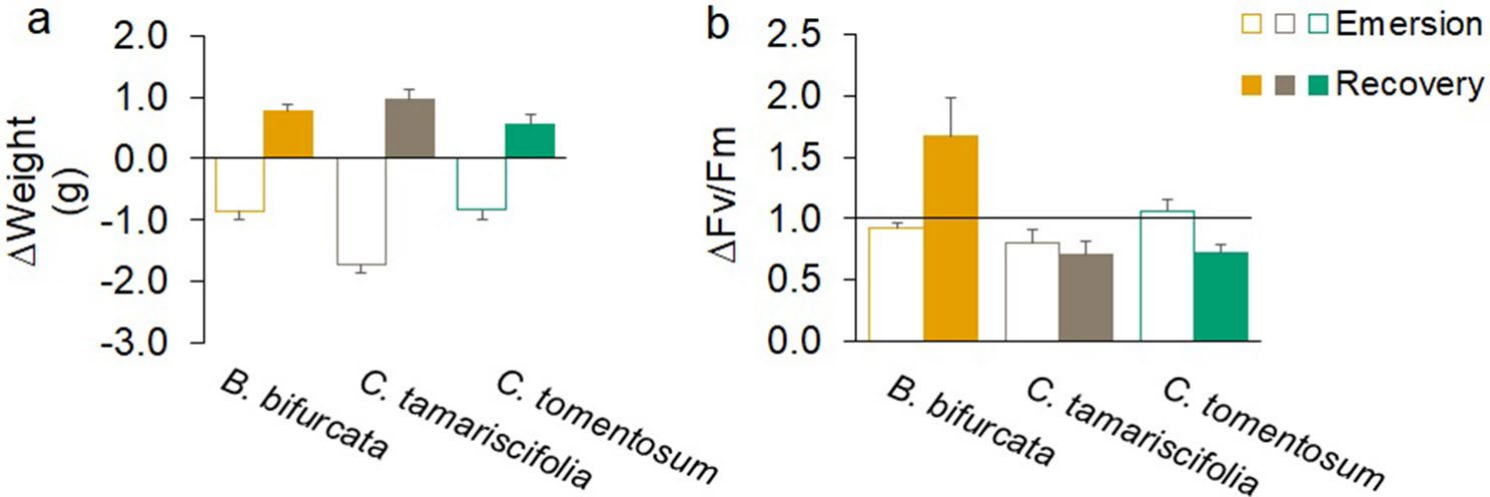
Mean (+ SE) value of (**a**) Δ weight, (**b**) Δ F_v_/F_m_ after emersion and recovery in the field measurements.

The weight of the fronds of all three species decreased with increasing air temperature during emersion, although the fronds were not completely dry. After immersion and recovery for 30 min, the weight increased significantly (GEEs: *B. bifurcata:* χ^2^ = 120, df = 1, *p* < 0.001; *C. tamariscifolia*: χ^2^ = 245, df = 1, *p* < 0.001; *C. tomentosum:* χ^2^ = 15, df = 1, *p* < 0.001) (Fig. 1a), although there were differences between species due to greater weight loss in *C. tamariscifolia*.

The variation in F_v_/F_m_ (ΔF_v_/F_m_) differed in magnitude between the emersion and recovery periods for *B. bifurcata* (GEE: χ^2^ = 6.61, df = 1, *p* = 0.010, n = 10) and *C. tomentosum* (GEE: χ^2^ = 111.30, df = 1, *p* < 0.001, n = 10), but not for *C. tamariscifolia* (GEE: χ^2^ = 0.54, df = 1, *p* = 0.460, n = 10). After emersion, the change in F_v_/F_m_ was slightly lower than 1 in *B. bifurcata* and *C. tamariscifolia*, whereas in *C. tomentosum* the values of the ratio were above 1 (Fig. 1b). After recovery, *B. bifurcata* showed the greatest change in ΔF_v_/F_m_ and the value was higher than 1, whereas in *C. tomentosum* the change was smaller and resulted in a reduction in F_v_/F_m_ (Fig. 1b).

### Laboratory experiment

#### Environmental conditions

Light intensity in the immersion tanks was 195 ± 5 μmol photon·m^-2^ s^−1^ and there were no differences between treatments (GLM: χ^2^ = 7.68, df = 2, *p* = 0.020, n = 6). The experimental seawater temperature was 18.45 ± 0.01 °C in control treatments, 19.74 ± 0.02 °C in the marine heatwave treatments and 21.77 ± 0.03 °C in the extreme marine heatwave treatments. Temperatures differed significantly between treatments (GLM: χ^2^ = 14,621, df = 2, *p* < 0.001, n = 6).

During the four hours of emersion in the tanks, the air temperature remained constant in the control treatment, whereas it increased gradually in the atmospheric heatwave and extreme atmospheric heat wave treatments. After the fourth hour in the two emersion cycles, when target temperatures were reached, the temperature under the macroalgal canopies was 22.93 ± 0.08 °C in the control treatment, 28.40 ± 0.19 °C in the heatwave treatment and 30.60 ± 0.14 °C in the extreme heatwave treatment.

The humidity (%) below the canopies after the fourth hour of emersion was 96.15 ± 0.15 in the control treatment, 93.91 ± 0.48 in the heatwave treatment and 91.44 ± 0.90 in the extreme heatwave treatment. The temperatures and the humidity were significantly different among treatments (GLM for temperature: χ^2^ = 1458.40, df = 2, *p* < 0.001, n = 6) (GLM for humidity: χ^2^ = 32.36, df = 2, *p* < 0.001, n = 6) (Supplementary Fig. S3).

#### Mortality

**Figure 2:**
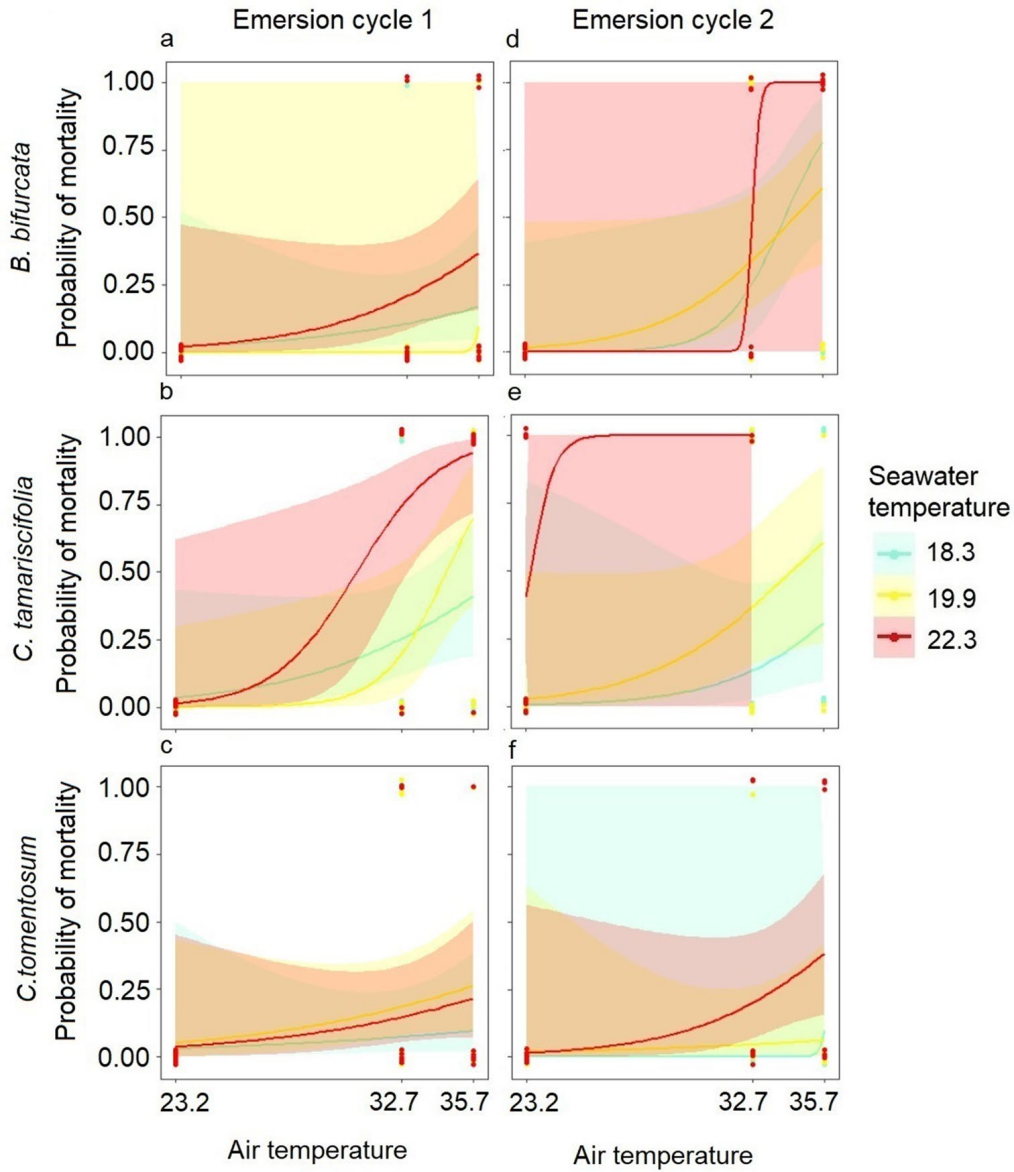
Mortality probability in relation to air and seawater temperature after each emersion cycle in the laboratory experiment. Curves are fitted by GLMs with a binomial error structure and a confidence interval of 95%. Dots indicate binary mortality observations in each treatment (air temperature, control ~ 23.2 °C, atmospheric heatwave ~ 32.7 °C and extreme atmospheric heatwave ~ 35.7 °C), (seawater temperature, control ~ 18.3 °C, marine heatwave ~ 19.9 °C and extreme marine heatwave ~ 22.3 °C).

After the first emersion cycle, dead fronds of all species were observed in the atmospheric heatwave and extreme atmospheric heatwave treatments, although the mortality rates differed between species. The probability curves for mortality in *B. bifurcata* and *C. tomentosum* were similar and showed a lower probability of mortality than those of *C. tamariscifolia* due to the increase in air temperature (Fig. 2a–c). Seawater temperature also had a significant additive effect on the mortality of *C. tamariscifolia* (Table 1) with a significant increase in the probability of mortality in the extreme marine heatwave treatment.

**Table 1:** The summarised results of GLMs used to test the effects of seawater (ST) and air temperature (AT) on the mortality, growth and ΔF_v_/F_m_ of fronds after the emersion cycles and during the recovery period, in the laboratory experiment.

	Emersion cycle 1			Emersion cycle 2			Recovery		
	χ^2^	df	*p*	χ^2^	df	*p*	χ^2^	df	*p*
**Mortality**									
*B. bifurcata*									
ST	4.49	2	0.106	0.69	2	0.707	n.a.		
AT	9.01	2	0.011*	43.38	2	**< 0.001**	n.a.		
STxAT	1.84	4	0.766	3.95	4	0.413	n.a.		
*C. tamariscifolia*									
ST	12.65	2	**< 0.010**	15.54	2	**< 0.001**	n.a.		
AT	42.07	2	**< 0.001**	13.23	1	**< 0.001**	n.a.		
STxAT	3.82	4	0.430	0.00	2	1	n.a.		
*C. tomentosum*									
ST	1.55	2	0.461	4.81	2	0.091	n.a.		
AT	15.49	2	**< 0.001**	7.09	2	0.029*	n.a.		
STxAT	0.84	4	0.932	3.20	4	0.524	n.a.		
**Growth**									
*B. bifurcata*									
ST	3.72	2	0.156	0.35	2	0.838	24.39	2	**< 0.001**
AT	58.96	2	**< 0.001**	65.99	1	**< 0.001**	18.07	1	**< 0.001**
STxAT	5.48	4	0.241	2.76	2	0.251	8.00	2	0.018*
*C. tamariscifolia*									
ST	1.53	2	0.465	7.24	2	0.027*	2.39	2	0.302
*C. tomentosum*									
ST	1.75	2	0.417	0.63	2	0.729	14.65	2	**< 0.001**
AT	60.19	2	**< 0.001**	58.48	2	**< 0.001**	21.22	2	**< 0.001**
STxAT	5.07	4	0.279	1.25	4	0.869	3.96	4	0.411
**ΔF**_**v**_**/F**_**m**_									
*B. bifurcata*									
ST	2.35	2	0.309	0.04	2	0.980	0.97	2	0.616
AT	6.33	2	0.042*	0.69	1	0.405	4.02	1	0.045*
STxAT	8.58	4	0.072	0.69	2	0.706	2.59	2	0.274
*C. tamariscifolia*									
ST	7.27	2	0.026*	0.87	2	0.648	0.64	2	0.723
*C. tomentosum*									
ST	2.52	2	0.283	0.19	2	0.907	2.08	2	0.353
AT	0.08	2	0.962	11.47	2	**0.003**	0.49	2	0.782
STxAT	9.64	4	0.047*	2.83	4	0.587	8.41	4	0.078

Significant effects (*p* < 0.01) are indicated in bold. * indicates marginal significance. “n.a.” indicates not analysed as no mortality was recorded in the recovery period. Note that due to an insufficient number of replicates, the effect of the extreme atmospheric heatwave (35.7 °C) on the growth and the ΔF_v_/F_m_ of *B. bifurcata* after the second emersion cycle was not modelled. Likewise, the effect of air temperature on the growth and the ΔF_v_/F_m_ of *C. tamariscifolia* after the cycles of emersion were not modelled.

After the second emersion cycle under the control air temperature, dead fronds of *C. tamariscifolia*, but not of *B. bifurcata* or *C. tomentosum*, were observed (Fig. 2d–f). Under the atmospheric heatwave and extreme atmospheric heatwave treatments, the increase in air temperature had a significant effect on *B. bifurcata* mortality, which was much higher in the second than in the first emersion cycle, indicating cumulative effects (Fig. 2d). As in the first emersion cycle, seawater and air temperatures had a significant additive effect on *C. tamariscifolia* mortality (Table 1). The probability of mortality increased under the extreme marine heatwave conditions compared to the control, and it also increased when air temperature increased in the atmospheric heat wave treatment, indicating cumulative effects in the second emersion cycle (Fig. 2e). Air temperature had a marginal effect on *C. tomentosum* mortality (Table 1, Fig. 2f). No dead fronds of any of the species were recorded after the recovery period.

#### Growth

**Figure 3:**
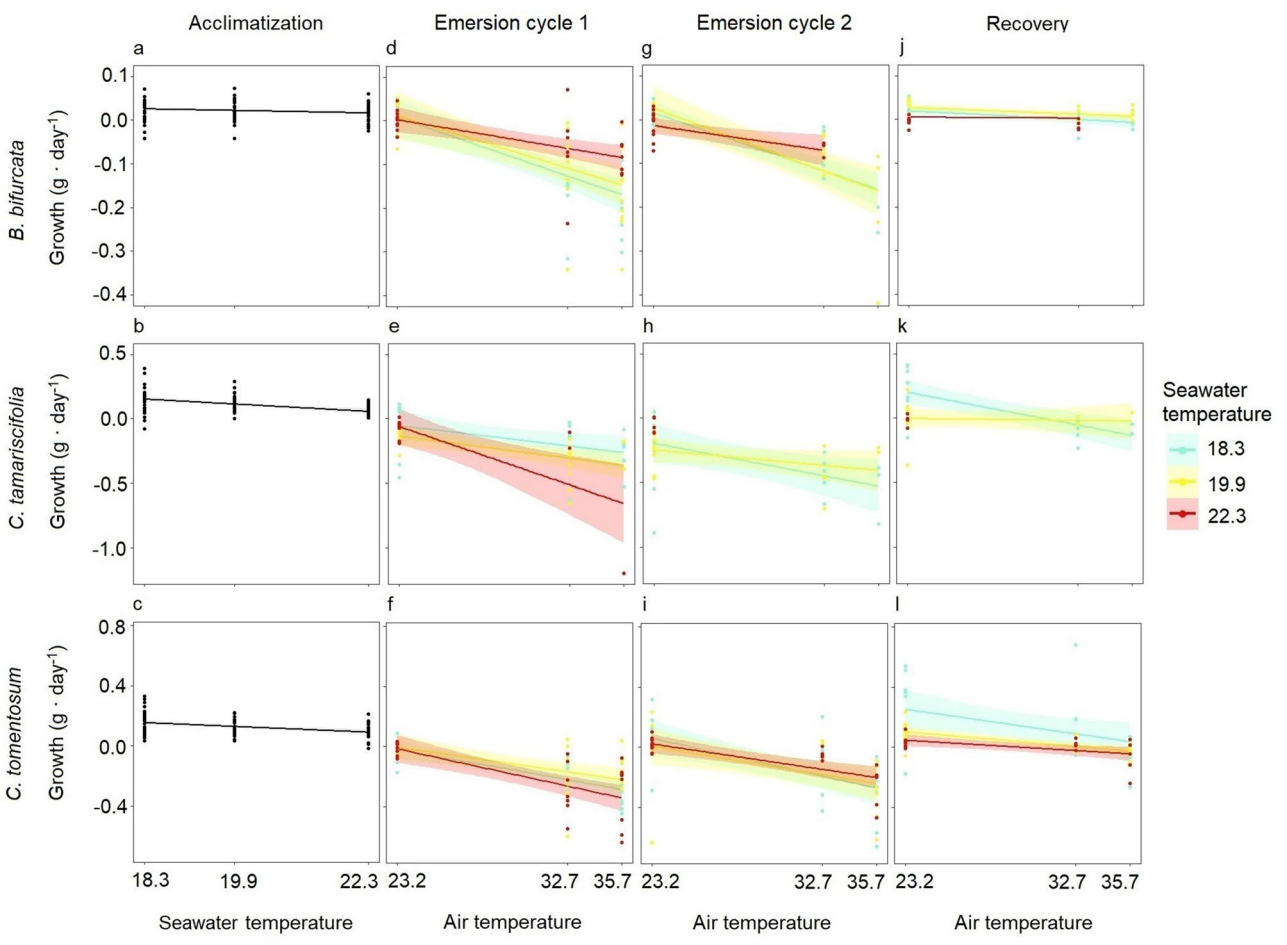
Trends in macroalgal growth as a function of temperature treatments (air temperature: control ~ 23.2 °C, atmospheric heatwave ~ 32.7 °C and extreme atmospheric heatwave ~ 35.7 °C; seawater temperature: control ~ 18.3 °C, marine heatwave ~ 19.9 °C and extreme marine heatwave ~ 22.3 °C) after acclimatization to seawater temperature (**a**–**c**), after emersion cycles (**d**–**i**) and after recovery (**j**–**l**) in the laboratory experiment. The points represent replicates and the smoothed curves are the data fitted by GLMs with a confidence interval of 95%. Note that due to an insufficient number of replicates, the effect of the highest air temperature (35.7 °C) on the growth of *B. bifurcata* after the second emersion cycle was not formally modelled. Likewise, the effect of air temperature on the growth of *C. tamariscifolia* after the two emersion cycles was not formally modelled.

During the 12-day period of acclimatization to the experimental seawater temperatures, the growth of *C. tamariscifolia* and *C. tomentosum* was greater than that of *B. bifurcata* (Fig. 3a-c)*.* Growth of the three species decreased significantly as the seawater temperature increased (GLMs: *B. bifurcata*: χ^2^ = 7.58, df = 2, *p* < 0.05, n = 30; *C. tamariscifolia*: χ^2^ = 32.46, df = 2, *p* < 0.001, n = 30; *C. tomentosum*: χ^2^ = 21.37, df = 2, *p* < 0.001, n = 30).

After the first emersion cycle, the increase in air temperature significantly affected the growth of *B. bifurcata* and *C. tomentosum* (Table 1). At the control air temperature, the weight of *C. tamariscifolia* decreased, whereas the weight of *B. bifurcata* and *C. tomentosum* remained unchanged. In the atmospheric heatwave and extreme atmospheric heatwave treatments, the weight of all three species decreased, with *C. tamariscifolia* showing the greatest decrease, followed by *C. tomentosum* and *B. bifurcata* (Fig. 3d–f). Because of the high mortality rate, which resulted in an insufficient number of replicates, the effect of air temperature on the growth of *C. tamariscifolia* could not be modelled, although the trend was for a marked decrease in growth.

After the second emersion cycle, the growth of *B. bifurcata* and *C. tomentosum* decreased significantly as the air temperature increased (Table 1). Growth of the three species at the control air temperature was similar to that observed after the first emersion cycle. In the atmospheric heatwave and extreme atmospheric heatwave treatments, the greatest decrease in growth occurred in *C. tamariscifolia*, followed by *C. tomentosum* and *B. bifurcata* (Fig. 3g–i)*.* The increase in air temperature during consecutive emersion cycles had a cumulative negative effect on the growth of *C. tamariscifolia*. This trend was clearly evidenced in those fronds at the control seawater temperature (18.3 °C), which presented a steeper negative slope after the second emersion (Fig. 3e,h).

After the recovery period, the fronds of *B. bifurcata* and *C. tomentosum* subjected to an increase in water temperature and previously exposed to an increase in air temperature still showed a significant additive effect that led to a decrease in growth (Table 1). Although the fronds of *C. tamariscifolia* and *C. tomentosum* in the control seawater temperature treatment grew, the growth did not compensate for the previous weight loss during the emersion stress. The weight of *C. tamariscifolia* and *C. tomentosum* exposed to the extreme marine heatwave was still lower after the recovery period (Fig. 3j–l).

#### *∆F*_*v*_*/F*_*m*_

**Figure 4:**
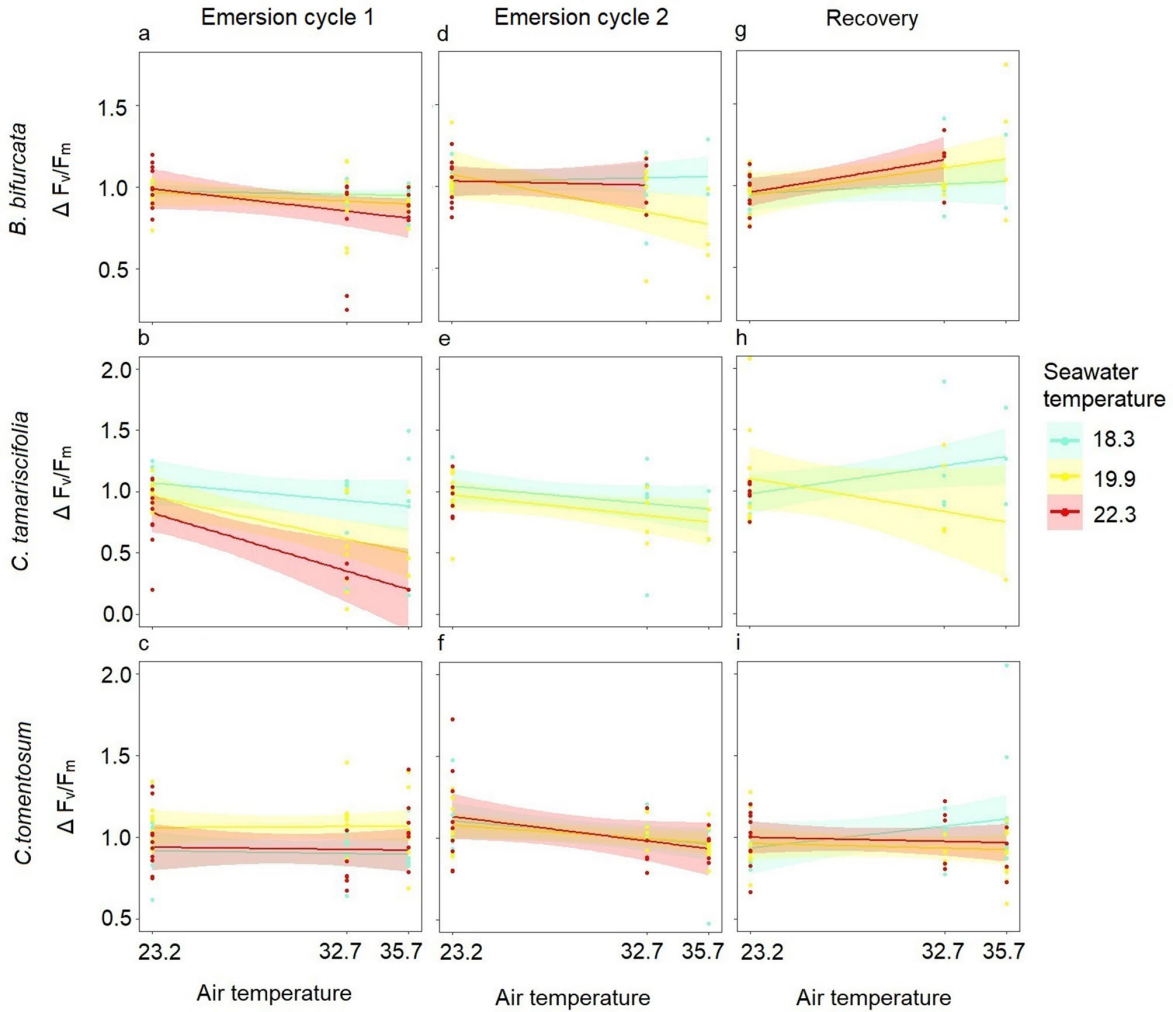
Trends in Δ F_v_/F_m_ as a function of temperature treatments (air temperature: control ~ 23.2 °C, atmospheric heatwave ~ 32.7 °C and extreme atmospheric heatwave ~ 35.7 °C; seawater temperature: control ~ 18.3 °C, marine heatwave ~ 19.9 °C and extreme marine heatwave ~ 22.3 °C) after the emersion cycles (**a**–**f**) and recovery periods (**g**–**i**) in the laboratory experiment. The points are the replicates and the smoothed curves are the data fitted by GLMs with a confidence interval of 95%. Note that due to an insufficient number of replicates, the effect of the highest air temperature level (35.7 °C) on the ∆F_v_/F_m_ of *B. bifurcata* after the second emersion cycle was not formally modelled. Likewise, the effect of air temperature on the ∆F_v_/F_m_ of *C. tamariscifolia* after the two emersion cycles was not formally modelled.

The photosynthetic performance of the three species decreased when the fronds were subjected to atmospheric heatwave and extreme atmospheric heatwave treatments, as shown by the change in F_v_/F_m_, with values well below 1, especially in *C. tamariscifolia* (Fig. 4a–f). After the first emersion cycle, under control air temperature, the change in F_v_/F_m_ was similar in *B. bifurcata* and *C. tomentosum* and was slightly higher in *C. tamariscifolia*, with a value below 1. Under atmospheric heatwave and extreme atmospheric heatwave treatments, the change in F_v_/F_m_ was minimal in *C. tomentosum* (ΔF_v_/F_m_ ~ 1), and the ΔF_v_/F_m_ changed very little in *B. bifurcata*, to values slightly below 1. *Cystoseira tamariscifolia* showed the most marked variation in ΔF_v_/F_m,_ (Fig. 4b), with the F_v_/F_m_ being markedly lower after emersion than prior to emersion. The effect of extreme atmospheric heatwave on the ΔF_v_/F_m_ in *C. tamariscifolia* could not be modelled, because of the insufficient number of replicates caused by the high mortality. However, the increase in seawater temperature had a marginally significant effect on the variation in ΔF_v_/F_m_ in *C. tamariscifolia*, for which values were below 1 in the marine heatwave and extreme marine heatwave treatments (Table 1, Fig. 4b).

After the second emersion cycle at control air temperature, the values of ΔF_v_/F_m_ in *C. tomentosum* and *B. bifurcata* were higher than 1, indicating an increase relative to the pre-emersion values, whereas the ΔF_v_/F_m_ in fronds of *C. tamariscifolia* was ~ 1, indicating no variation in F_v_/F_m_. Under atmospheric heatwave and extreme atmospheric heatwave treatments, the ΔF_v_/F_m_ tended to change in the three species, to values below 1, when air temperature increased (Fig. 4d–f), although this factor only significantly affected the ΔF_v_/F_m_ of *C. tomentosum* (Table 1). The consecutive exposure to high air temperature had a cumulative effect on the decrease in photosynthetic performance in *C. tomentosum* (Fig. 4f).

After recovery in immersion tanks, at control seawater temperature, ∆F_v_/F_m_ varied notably in fronds of *C. tamariscifolia*, indicating that after recovery, the F_v_/F_m_ values exceeded those values before this period, whereas ∆F_v_/F_m_ in *B. bifurcata* and *C. tomentosum* remained around 1, indicating no recovery of the F_v_/F_m_ values (Fig. 4 g–i). In the marine heatwave treatment (~ 19.9 °C), the ∆F_v_/F_m_ in *C. tamariscifolia* fronds differed markedly from that in fronds in control seawater, implying the absence of recovery and additional loss of F_v_/F_m_. By contrast, in marine heatwave and extreme marine heatwave conditions, ∆F_v_/F_m_ scarcely varied in *C. tomentosum* and increased only slightly in *B. bifurcata* (Fig. 4g,i)*.* The overall change in F_v_/F_m_ after recovery was slight, and the greatest increase (1.5-fold) appeared in *C. tamariscifolia* previously subjected to atmospheric heatwave conditions (air temperature = 32.7 °C).

#### C:N ratio

**Figure 5:**
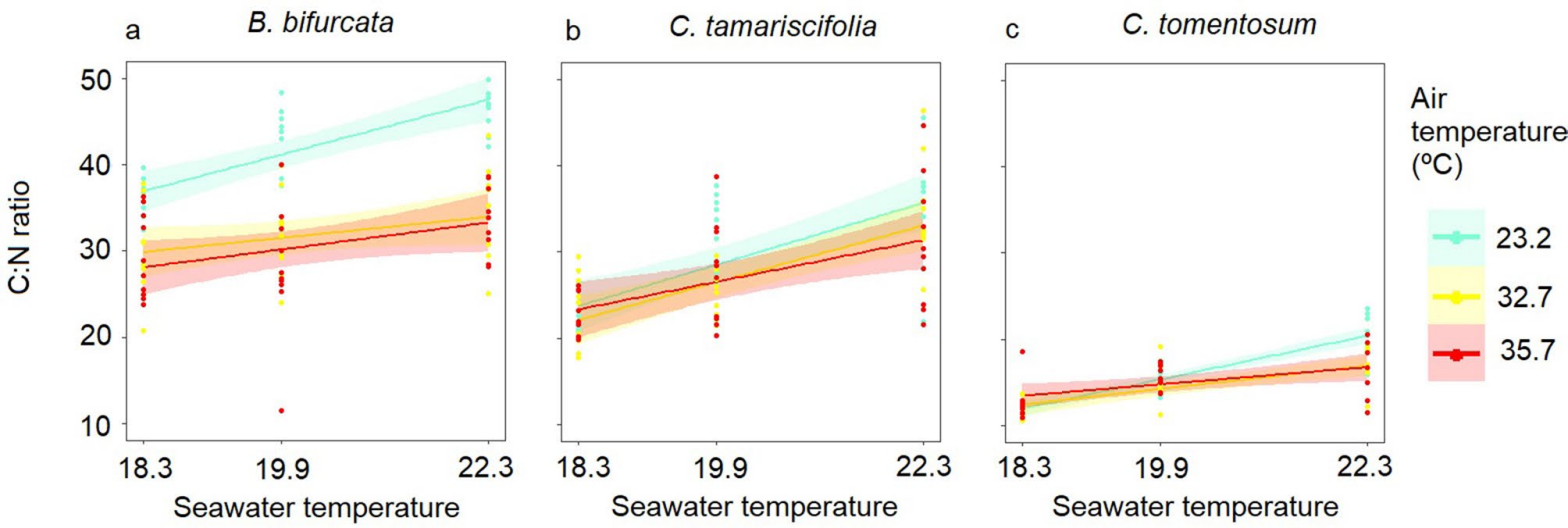
C:N ratio as a function of seawater temperature treatments (control ~ 18.3 °C, marine heatwave ~ 19.9 °C and extreme marine heatwave ~ 22.3 °C) in fronds exposed to control (~ 23.2 °C), atmospheric heatwave (~ 32.7 °C) and extreme atmospheric heatwave (~ 35.7 °C) air temperatures during emersion. The points are the replicates and the smoothed curves are the data fitted by GLMs with a confidence interval of 95%.

The C:N ratio was highest in fronds of *B. bifurcata,* followed by those of *C. tamariscifolia* and *C. tomentosum* (Fig. 5)*.* In general, the C:N ratio increased as the seawater temperature increased (Table 2, Fig. 5), whereas air temperature had only a marginally significant effect on the C:N ratio in *B. bifurcata.* More specifically, the increase in seawater temperature led to a significant decrease in %N in all three macroalgae (Table 2, Fig S4). By contrast, the increase in air temperature tended to decrease the %C in *B. bifurcata* and *C. tomentosum* (Table 2, Supplementary Fig. S4). Air and seawater temperature interacted in the C:N ratio in *C. tomentosum* as the C:N ratio tended to increase as the seawater temperature increased, although the trend was less marked in fronds exposed to higher air temperature during emersion cycles (note the less steep slopes for air temperatures of 32.7 and 35.7 °C in Fig. 5c).

**Table 2:** The summarised results of GLMs testing the effects of seawater (ST) and air (AT) temperature on the C:N ratio, C% and N% of the fronds after the low tides and the recovery in the laboratory experiment.

	*B. bifurcata*			*C. tamariscifolia*			*C. tomentosum*		
	χ^2^	df	*p*	χ^2^	df	*p*	χ^2^	df	*p*
***C:N ratio***									
ST	28.26	2	**< 0.001**	33.81	2	**< 0.001**	96.19	2	**< 0.001**
AT	8.15	2	0.017*	0.50	2	0.777	2.01	2	0.366
ST x AT	13.04	4	0.011*	9.91	4	0.042	18.11	4	**0.001**
***C %***									
ST	6.48	2	0.039*	9.48	2	**< 0.01**	2.27	2	0.321
AT	67.35	2	**< 0.001**	0.86	2	0.651	7.25	2	0.026*
ST x AT	4.49	4	0.344	1.51	4	0.827	5.81	4	0.214
***N %***									
ST	9.77	2	**0.007**	53.29	2	**< 0.001**	93.99	2	**< 0.001**
AT	16.99	2	**< 0.001**	1.62	2	0.446	2.68	2	0.261
ST x AT	6.57	4	0.160	8.84	4	0.065	14.14	4	**0.007**

Significant effects (*p* < 0.01) are shown in bold. * indicates marginal significance.

## Discussion

This study showed that the stressful conditions created particularly by atmospheric heatwaves and, to less extent, by marine heatwaves negatively affected the survival and physiology of intertidal macroalgae after exposure to two consecutive emersion cycles, and that these stressors had an additive effect. Increased air temperature caused important negative short- and intermediate-term effects on the physiological performance of the macroalgae in the field and the laboratory experiment, effects that were cumulative after exposure to two consecutive emersion cycles in the laboratory experiment. This pattern is consistent with the findings of other studies in which air temperature was found to be a relevant factor modulating physiological stress in intertidal macroalgae^6,15,20,26,37^ and of others that related contractions in the distribution of intertidal macroalgae to large increases in air temperature^37,52,53^. The seawater temperatures applied in the laboratory experiment (which reflected realistic present and future climatic scenarios according to historic databases, scientific literature and IPCC predictions) were below the physiological threshold of 23 °C for *B. bifurcata*^54^. The highest temperature was above the optimum for net photosynthesis in *B. bifurcata* and *C. tamariscifolia* (20.56 ± 1.94 °C and 21.9 ± 1.13 °C, respectively)^55^. Thus, the increased seawater temperatures negatively affected the physiological performance of fronds, but led to sub-lethal effects.

The different indicators measured in macroalgae after the emersion cycles showed a consistent decline of the physiological performance caused by atmospheric and marine heatwaves, which was greater under the most extreme conditions, i.e. global warming scenario. The F_v_/F_m_ ratio reflected short term regulatory mechanisms such as photoprotection^56^, whereas the changes in growth and the C:N ratio integrated physiological processes over longer periods^48^. Values of ΔF_v_/F_m_ much lower than 1, caused by the high air temperature, suggest short-term regulatory effects on the physiology of macroalgae and were probably a consequence of desiccation and thermal stress. Tissue desiccation tends to decrease the F_v_/F_m,_ due to a diminution of osmotic potential^18,49,52,57,58^, affects photosynthesis, C fixation and generates overproduction of reactive oxygen species (ROS)^23,59,60^ with negative consequences on physiological processes^23,57,59,61^, and the high temperatures also cause enzyme denaturalization^59,62^. The macroalgae resist this disruptive stress and prevent lethal damage by reducing all metabolic activities^63,64^, thereby inhibiting growth^49^. This capacity was evidenced by the survival of the large proportion of fronds that suffered severe desiccation and also the increased weight and F_v_/F_m_ observed after recovery. The increasing seawater temperature also increased significantly the C:N ratio, as previously observed in diverse red and brown seaweeds^50^. This increase occurred as a consequence of significant decreases in %N (Table 2, Supplementary Figure S4) coincident with patterns observed in laminarians^65^. Higher seawater temperatures have often been linked to a decrease in the activity of nitrate reductase in intertidal and subtidal macroalgae^66,67^, implying a lower assimilation capacity of NO_3_^−^ and lower incorporation rates of N. The above-mentioned effects, together with the cumulative stress caused by two consecutive cycles of emersion under atmospheric heatwaves, probably overwhelmed the shelf-shading protective mechanisms of the canopies against desiccation^6,13,14,52,68^, the production of heat shock proteins and the repair processes^10,23,48,59^, and prevented the fronds from recovering the pre-stress F_v_/F_m_ and weight, leading to death in some cases as evidenced by the cumulative mortality in *C. tamariscifolia* and *B. bifurcata*. This may result in a decline of the abundance of these habitat-forming macroalgae in the intertidal, as reported in^37^.

The species under study showed different capacities to tolerate increase in air temperature, with *C. tamariscifolia* being the most vulnerable and *C. tomentosum* the most resistant and resilient. The high resilience of *C. tomentosum* was indicated by the recovery of F_v_/F_m_ to pre-stress values, although the second emersion weakened this capacity suggesting cumulative stress. The decrease in F_v_/F_m_ in *B. bifurcata* after emersion in the field was followed by a large increase in F_v_/F_m_ after the recovery period, revealing the resilience of this alga after moderate desiccation. Moreover, in *C. tomentosum* and *B. bifurcata*, the F_v_/F_m_ changed little, with values slightly higher than 1 obtained after the second laboratory emersion experiment at control air temperature. This suggests that after moderate desiccation, the water loss was not high enough to alter the efficiency of charge separation during photosynthesis^18,58^, indicating the absence of a high level of physiological stress. The morphological traits also influence resistance to desiccation^68^. The desiccation rate is lower in the thick and voluminous fronds of *C. tomentosum,* which have a smaller specific surface than the fronds of *C. tamariscifolia*^10,69^. In comparison to *C. tamariscifolia*, the less pronounced decrease in growth observed in *B. bifurcata* after the two emersion cycles was mainly a consequence of a slower growth rate^51^. The different resistance and resilience to increasing air temperatures might imply a change in the structure of macroalgal assemblages on intertidal rocky shores of NW Spain under global warming scenarios, with an increase in the relative abundance of *C. tomentosum* and *B. bifurcata* and a decrease in *C. tamariscifolia*.

Although experimental and field responses showed similar patterns, extrapolation of laboratory results to the field must be done with caution. In the laboratory experiment, the humidity levels were higher than in the intertidal, which implies that emersions under the same conditions at the intertidal could lead to stronger negative effects such as higher desiccation, lower physiological performance and greater mortality. By contrast, the irradiance levels in the laboratory were much lower than those experienced in the intertidal, and might have been below the saturation points (I_k_) of the species, as for example the I_k_ of the intertidal fucoid *Fucus spiralis* is above 300 μmol photon m^−2^ s^−1^ under emersion with air temperatures of 20°C^22^. This light limitation might result in lower photosynthetic efficiency and smaller growth rates during the laboratory experiment with potentially more intense negative effects of the desiccation during emersions^22^ and inhibition of the recovery responses during immersions (see^70^). Nevertheless, our results might have broader consequences for the future geographical distribution of macroalgae. The NW Iberian Peninsula represents the range center of the species studied (www.marinespecies.org; www.obis.org)^71^. In this area, previous studies have predicted an expansion and increase in the abundance of *B. bifurcata, C. tamariscifolia* and *Codium* spp. in response to the increased sea surface temperature expected under global warming scenarios (see Introduction). However, the present findings show that atmospheric heatwaves during low tide may be detrimental to the performance and survival of these species, probably inhibiting the predicted shifts. The importance of air temperature for the habitat suitability of intertidal macroalgae is evident; however, species distribution models (SDMs) and databases that take air temperature into account^72–74^ are scarcer than models including only seawater temperature. To improve the forecasting of potential range shifts in intertidal macroalgae due to global warming, air temperature should be included as a factor in SDMs. Air and seawater temperature acted additively increasing the mortality of *C. tamariscifolia* after emersion cycles and decreasing the growth of *B. bifurcata* and *C. tomentosum* after a period of recovery. These additive effects coincide with patterns observed in other intertidal macroalgae^20^, probably because both factors affect similar physiological mechanisms^75^.

The fact that the macroalgae exposed to atmospheric heatwaves during two consecutive cycles of emersion suffered cumulative effects on their physiological performance and mortality is important, as air temperature and the frequency of heatwaves are expected to increase under global warming scenarios predicted for the region^8,9^. A decline in these bio-engineering macroalgae may remove habitat and shelter for several species in higher trophic levels, degrading the benthic community and having negative consequences on diversity and fisheries^33^. Future experiments could apply higher-resolution air temperature gradients to intertidal macroalgae to obtain data for constructing thermal physiological performance curves, especially under consecutive periods of exposure at low tide. Despite the limitations of laboratory experiments, their contribution to assessing the cumulative effects of chronic exposure to atmospheric heatwaves during low tide is crucial, as the findings can help to improve predictive models that forecast the effects of global warming on the physiology and distribution of ecologically important intertidal macroalgae.

## Materials and methods

### Field measurements

**Figure 6:**
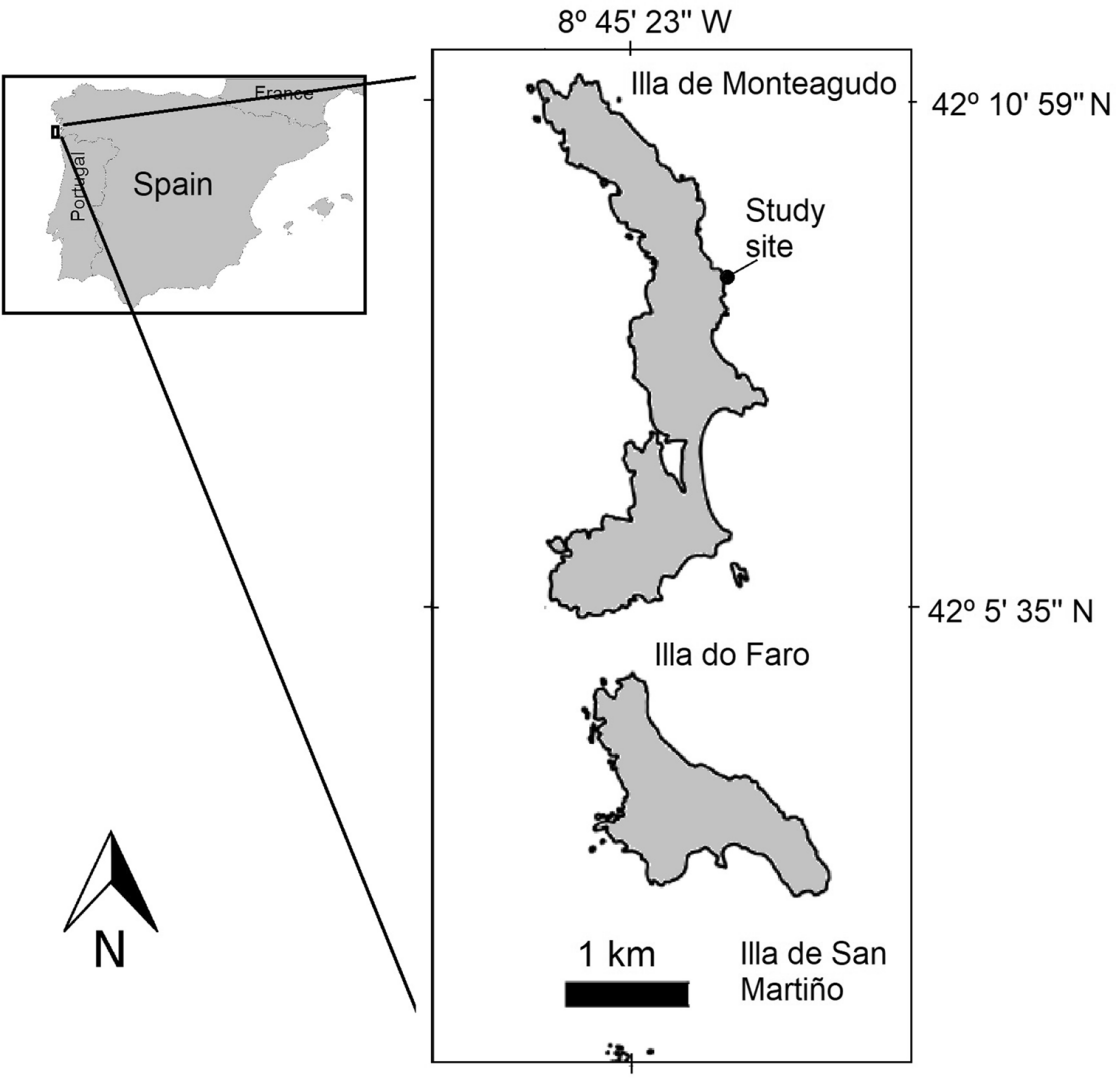
Location of the field measurements and the rocky intertidal sampling site on the Cíes Islands (Atlantic Islands of Galicia National Park, NW Spain). Map derived from BDLJE CC-BY 4.0 www.scne.es, modified with QGIS 3.4.5. software (www.qgis.org).

The field measurements were carried out on 4 August 2019 at noon during low spring tide to examine the effects of air temperature on the photosynthetic performance and wet weight of the co-occurring species *B. bifurcata*, *C. tamariscifolia* and *C. tomentosum* in situ. These species coexist at the middle intertidal zone on the Isle of Monteagudo (area surrounding coordinates 42.23551°N, 8.89956°W), which belongs to the Cíes Islands archipelago, which forms part of the Atlantic Islands of Galicia National Park (NW Iberian Peninsula; Fig. 6). The study site is located in the Ría de Vigo, which experiences mesotidal semi-diurnal tides. At this site, the target species cover wide areas of the mid intertidal zone, forming dense, bushy canopies that emerge during an average of 4 h under low spring tides under favourable conditions (high atmospheric pressure and low wave height).

On the day that the field measurements were conducted, the insolation was 76.8%, the average wind speed was 6 km/h, and the average air temperature was 19 °C (www.meteogalicia.gal).

A HOBO Pendant temp/light data logger (Onset Computer Corp., Bourne, MA, USA) with an accuracy range of ± 0.53 °C and resolution of 0.14 °C placed on the bare rock recorded light and temperature data every 5 min. In addition, three temperature and humidity iButton Hygrochron data loggers (Maxim Integrated Products, Dallas Semiconductor, USA) with an accuracy range of ± 0.50 °C and resolution of 0.0625 °C placed below the canopy of each species (total N = 9) recorded data every minute.

The physiological responses of the macroalgae were assessed after emersion during low tide and subsequent recovery by immersion in seawater. Once the tide ebbed, 10 fronds of each species (N = 30) were cut from the canopies, numbered and weighed to determine the pre-stress wet weight. The fronds were then dark adapted with two layers of thick opaque plastic for 30 min to allow the complete oxidation of reaction centers for determining basal fluorescence (F_o_). The maximum quantum yield of photosynthesis (F_v_/F_m_) was then measured in the central part of the fronds with a Pulse-Amplitude-Modulation (PAM) chlorophyll fluorometer. The fronds were held under direct sunlight for two hours and then were dark-adapted for 30 min, and the F_v_/F_m_ and wet weight were again determined. The recovery treatment was applied by placing the fronds in a bucket filled with seawater and covered with opaque plastic for 30 min, before repeating wet weight and F_v_/F_m_ measurements.

The variation in photosynthetic performance (ΔF_v_/F_m_) was calculated using Eq. (1):

1 $$\Delta \frac{{{\text{F}}_{{\text{v}}} }}{{{\text{F}}_{{\text{m}}} }}{ = }\frac{{\left( {{\text{F}}_{{\text{v}}} {\text{/F}}_{{\text{m}}} } \right)_{{{\text{after}}\;{\text{treatment}}}} }}{{\left( {{\text{F}}_{{\text{v}}} {\text{/F}}_{{\text{m}}} } \right)_{{{\text{before}}\;{\text{treatment}}}} }}$$

This ratio was used to evaluate the physiological condition of macroalgae, where values > 1 indicate an increase in the F_v_/F_m_, i.e. improved performance, and values < 1 indicate a decrease in F_v_/F_m_, i.e. poorer performance after the treatments. The variation in weight (ΔWeight) indicated the potential for desiccative water loss under emersion in the field, which is linked to the different physiological responses of each species under desiccation stress. ΔWeight was calculated in the same way as ΔF_v_/F_m_.

### Laboratory experiment

A laboratory experiment was performed between 18 June and 26 July 2019 to assess the effects of air and seawater temperatures on the physiological performance of macroalgae. The experimental set-up included Species (3 levels: *B. bifurcata*, *C. tamariscifolia* and *C. tomentosum*), Seawater temperature (3 levels: control ~ 18.3 °C, marine heatwave ~ 19.9 °C and extreme marine heatwave ~ 22.3 °C) and Air temperature during the emersion periods (3 levels: control ~ 22.3 °C, atmospheric heatwave ~ 32.7 °C and extreme atmospheric heatwave ~ 35.7 °C) as fixed orthogonal factors (n = 10). The seawater and air temperature treatments used in this experiment were selected to simulate realistic present and future situations according to baseline scenarios in which the current trends in greenhouse gas emissions remain unaltered^7^.

Control seawater temperature was the average ambient summer temperature in July and August between 2009 and 2018 at the nearby Baiona coastal station in the Ría de Vigo (http://www.intecmar.gal/MultiParam/Default.aspx); the marine heatwave temperature was the average of values higher than the 95th percentile of the records; and the extreme marine heatwave temperature was the marine heatwave temperature under a future scenario of global warming, with a 2.4 °C increase for the Iberian upwelling in the 2070–2100 period^41^. The emersion air temperatures corresponded to average air temperatures at noon on the Cíes Islands in July and August between 2009 and 2018 (www.meteogalicia.gal). Control air temperature was the average summer temperature at noon; atmospheric heatwave temperature was the average temperature at noon above the 95th percentile, recorded on more than three days (www.aemet.es); and the extreme atmospheric heatwave temperature represented a heatwave under a global warming scenario of a 3.0 °C increase. This increase is the average calculated from the predictions of baseline scenarios for the period 2081–2100^7^.

A total of 270 vegetative fronds (90 fronds per each species) were collected in the middle intertidal zone (Fig. 6) and transported to the Estación de Ciencias Mariñas de Toralla (ECIMAT) facilities, which form part of the Marine Research Centre (CIM) of the University of Vigo (Spain). The fronds were acclimated for 6 days to laboratory conditions to enable a steady growth response to be reached. All fronds were numbered, attached to nylon strings inside two 100 L tanks and immersed in running filtered (50 μm) seawater at 16 °C under the ambient 15:9 photoperiod typical of June. To simulate the average seawater temperatures experienced in the region in summer (~ 18 °C), the temperature of the seawater in the tanks was increased gradually over three days, by decreasing the 16 °C water flux and increasing the 18 °C water flux.

After acclimatization at 18 °C, the fronds were placed in 35L tanks with an open circulating system within an isothermal walk-in chamber at ~ 20 °C. Each seawater temperature was assigned to two tanks (total N = 6 tanks). The desired seawater temperatures were achieved by heating the 18 °C seawater with titanium aquarium heaters (100 W) inside head tanks, which supplied the heated water to other tanks containing the fronds. Heated water was mixed with water at 18 °C, and both fluxes were gradually regulated to increase the temperature at a rate of 1 °C per day. The fronds were acclimatized to the seawater temperatures for 12 days. In each tank, 15 fronds of each species were randomly placed and exposed to summer photoperiod conditions of 15:9 h light: darkness (Supplementary Fig. S5). Light was supplied from above by LED lamps. To prevent any effects due to differences in light within the tanks, the fronds were moved to different positions in each tank every 2 days. To minimize any effects of the tank, fronds were moved every 5 days between tanks containing seawater at the same temperature.

After the acclimatization period, the fronds were subjected to a daily emersion period of 4 h (from 10:00 to 14:00) simulating low spring tides, during 3 consecutive days. The fronds were then subjected to an immersion period (8 days) simulating neap tides and then to another period of 3 consecutive days with a daily emersion of 4 h. After the second emersion period, fronds were again submerged in seawater for 6 days at the respective seawater temperatures to check their recovery. The laboratory experiment lasted for 38 days in total (Supplementary Fig. S6).

During emersion, fronds were transferred from the seawater tanks to tanks containing moistened granite tiles (natural rocky substrata of the algae in the field) placed on shelves inside the same chamber for 4 h, i.e. average emersion time experienced by macroalgae in the mid intertidal zone during spring tides at the sampling site. The fronds were distributed in layers (fronds covering other fronds) simulating their natural distribution in the field. The emersion tanks were lit from above by LED lamps, with the same light intensity and photoperiod as in the seawater tanks. The air temperature was increased gradually during emersion by using infrared ceramic heaters positioned over the tanks, in which the air was re-circulated with small battery-operated fans. The air temperature was regulated with digital temperature controllers positioned above the canopies. Temperature and humidity during emersion were monitored by data loggers positioned below the canopies.

The response variables mortality, wet weight and F_v_/F_m_ were measured in fronds before each emersion period and 20 h after each (days 18, 22, 29, 33) and after six days of recovery (day 38).

The numbers of dead fronds were recorded. Mortality was considered a binary response variable (1 = dead, 0 = alive). Wet weight was obtained in fronds previously blotted with paper to remove excess water from the surface. Growth was determined as the difference in wet weight 20 h after each treatment and the wet weight before each treatment (emersion cycles 1 and 2/recovery), divided by the number of days, according to^20^. This calculation enabled to directly assess if there was an increase in weight and net growth (positive values), no increase (zero), or decrease in fronds weight due to losses of tissue (negative values) after the emersions and the recovery. The F_v_/F_m_ was measured before emersion cycles and/or recovery and 20 h later, and the variation in photosynthetic performance (ΔF_v_/F_m_) was calculated using Eq. (1). The C:N ratio was determined in fronds removed from the tanks at the end of the experiment. The fronds were rinsed repeatedly to remove salts and then ground to produce a fine, homogenous powder. The C:N ratio was determined by CNHS elemental microanalysis (in a Fisons Carlo Erba EA1108 elemental analyser) at the analytical facilities of the University of Vigo (CACTI-UVIGO).

### Data analysis

The responses of each species to environmental factors in the field measurements and the laboratory experiment were analysed separately due to expected species-specific responses.

To determine whether the Δweight and ΔF_v_/F_m_ in species differed between treatments in the field, generalized estimating equation models (GEEs) were used, with Treatment (2 levels: emersion vs. recovery) as a fixed factor. GEEs estimate the regression parameters considering the correlation between observations in clustered data^76^. The GEEs had an autoregressive correlation structure and a Gaussian error distribution.

Between-species differences in temperature and humidity in the field were tested using a general linear model (GLM) including Species (3 levels: *B. bifurcata*, *C. tamariscifolia* and *C. tomentosum*) as a fixed factor. In the laboratory experiment, GLMs were also used to test for any differences in seawater temperature and light between the immersion tanks, as well as for differences in air temperature and humidity between the emersion tanks.

To determine whether mortality, growth, ΔF_v_/F_m_ and C:N ratio differed between treatments in the laboratory experiment, the GLMs included Air temperature (3 levels: control ~ 22.3 °C, atmospheric heatwave ~ 32.7 °C and extreme atmospheric heatwave ~ 35.7 °C) and Seawater temperature (3 levels: control ~ 18.3 °C, marine heatwave ~ 19.9 °C and extreme marine heatwave ~ 22.3 °C) as fixed orthogonal factors. Mortality data were fitted by GLMs with a binomial error structure, whereas growth, ΔF_v_/F_m_ and C:N ratio data were fitted by GLMs with a Gaussian error structure. The GLMs were only used when treatment groups (Air temperature x Seawater temperature) included at least 4 observations, because in some cases there was an insufficient number of replicates due to the high mortality rate. Therefore, the models did not include the effect of the highest air temperature (35.7 °C) on the growth and the ΔF_v_/F_m_ of *B. bifurcata* after the second emersion cycles or the effect of the air temperature factor on the growth or the ΔF_v_/F_m_ of *C. tamariscifolia* after the two emersion cycles.

When response variables did not fulfil the parametric assumptions, or when the model was unbalanced, a more conservative significance level of *p* = 0.01 was established. The goodness of fit of the models was assessed by graphically checking the error distribution, and the statistical significance of the models was calculated using Chi-square tests^77^. When models were significant, post hoc multiple comparison tests were conducted and Bonferroni *p* value corrections were applied. All data are reported as means ± S.E. Statistical analyses were performed with R 3.5.3 software, by using the “geepack” package^76^ for the GEEs and default system packages for the other tests.

## Supplementary information

Supplementary Informations.
