# Supplementary material for: Heatwaves during low tide are critical for the physiological performance of intertidal macroalgae under global warming scenarios
Source: Sci Rep. 2020 Dec 8;10:21408. doi: 10.1038/s41598-020-78526-5 (PMC7722886; doi:10.1038/s41598-020-78526-5)
Supplement: Supplementary file 1 — Supplementary Informations. [file 41598_2020_78526_MOESM1_ESM.docx]

**SUPPLEMENTARY INFORMATION**

*Title*

Heatwaves during low tide are critical for the physiological performance of intertidal macroalgae under global warming scenarios

*Author list*

Marta Román^12^, Salvador Román^12^, Elsa Vázquez^12^, Jesús Troncoso^12^, Celia Olabarria^12^

^
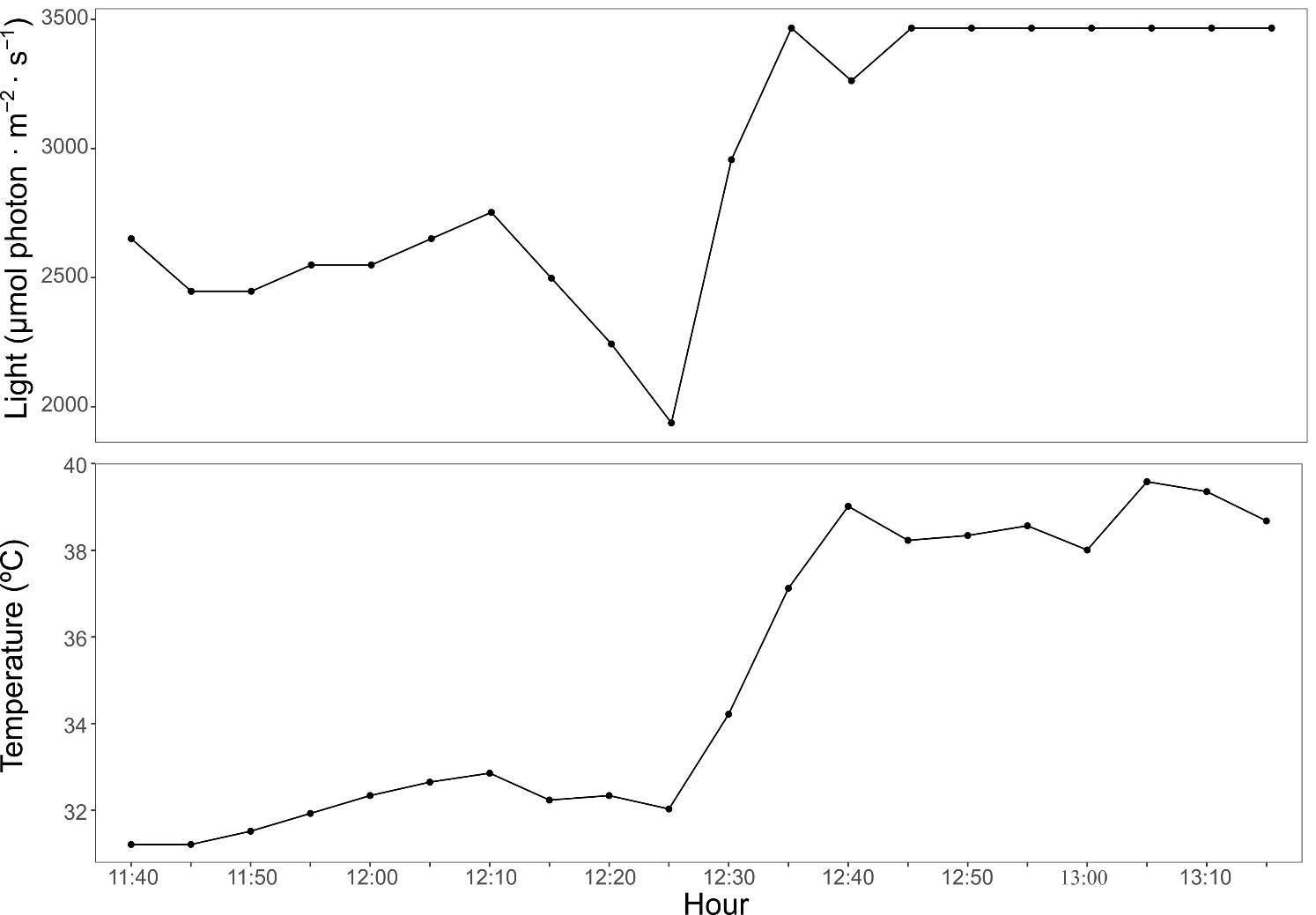
^

**SUPPLEMENTARY FIGURE S1**. Light and temperature profiles registered by a data logger placed on the bare rock during the field measurements.


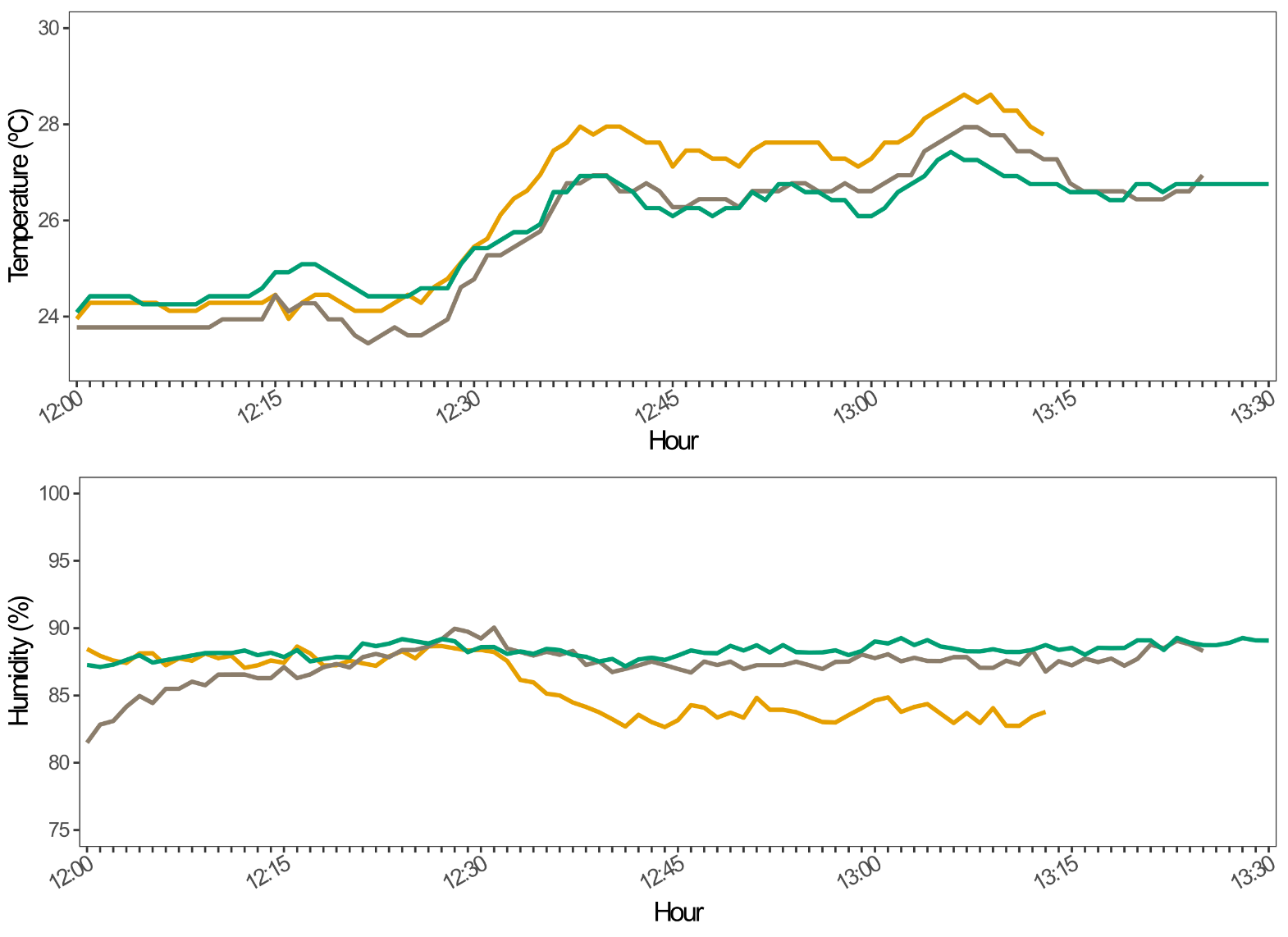


**SUPPLEMENTARY FIGURE S2**. Temperature and humidity profiles below the canopies of the three macroalgae during the field measurements. Maximum standard errors (±) for *B. bifurcata*, *C. tamariscifolia* and *C. tomentosum* (n=3), respectively: Temperature= 1.6, 1.4, 1.9; Humidity = 3.5, 2.9, 3.2.


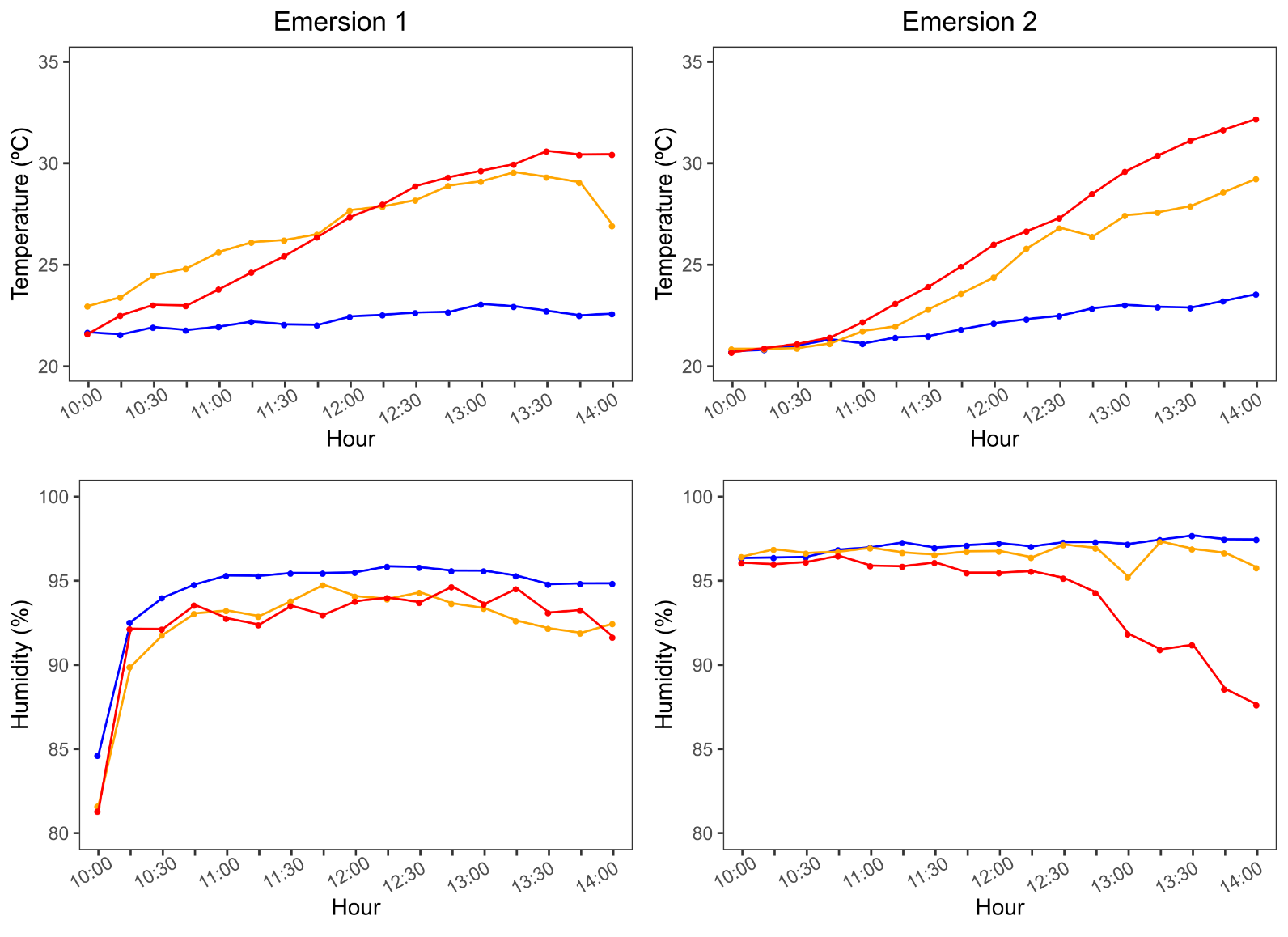


**SUPPLEMENTARY FIGURE S3**. Temperature and humidity profiles below the canopies of the three macroalgae during experimental emersion. The blue line represents the control emersion (~23.2ºC), the yellow line represents the emersion under atmospheric heatwave (~32.7ºC) and the red line represents the emersion under extreme atmospheric heatwave (~35.7ºC). Maximum standard errors (±) for control, atmospheric heatwave and extreme atmospheric heatwave emersion periods (n= 6), respectively: Temperature= 0.5, 1.5, 0.8; Humidity = 1.4, 3.9, 3.0.


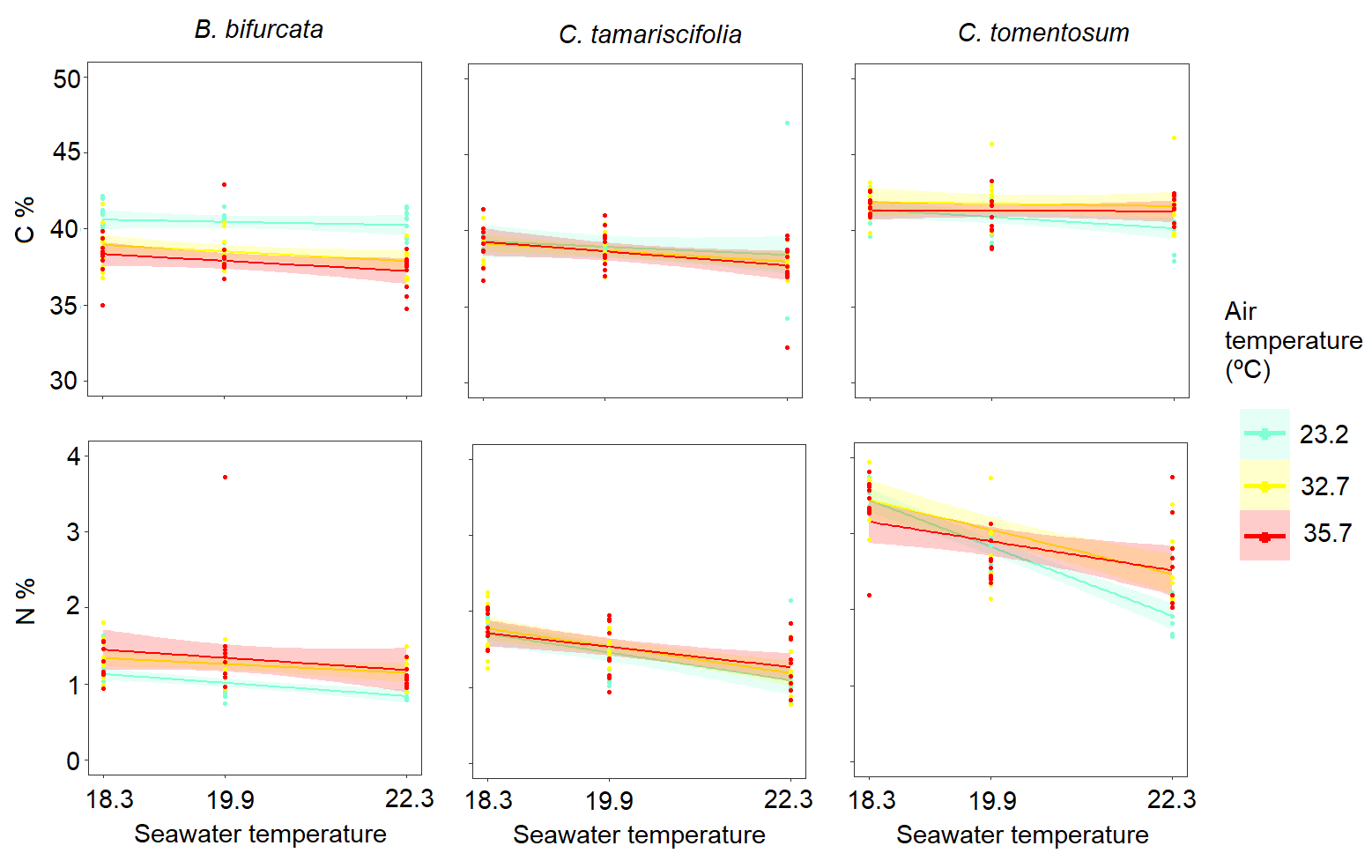


**SUPPLEMENTARY FIGURE S4.** C % and N % as a function of seawater temperature (control~ 18.3 ºC, marine heatwave~ 19.9 ºC and extreme marine heatwave~ 22.3 ºC) in fronds exposed to air temperature treatments (control ~23.2 ºC, atmospheric heatwave ~32.7 ºC and extreme atmospheric heatwave ~35.7 ºC) during emersion. The points are the replicates and the smoothed curves are the data fitted by GLMs with a confidence interval of 95%.


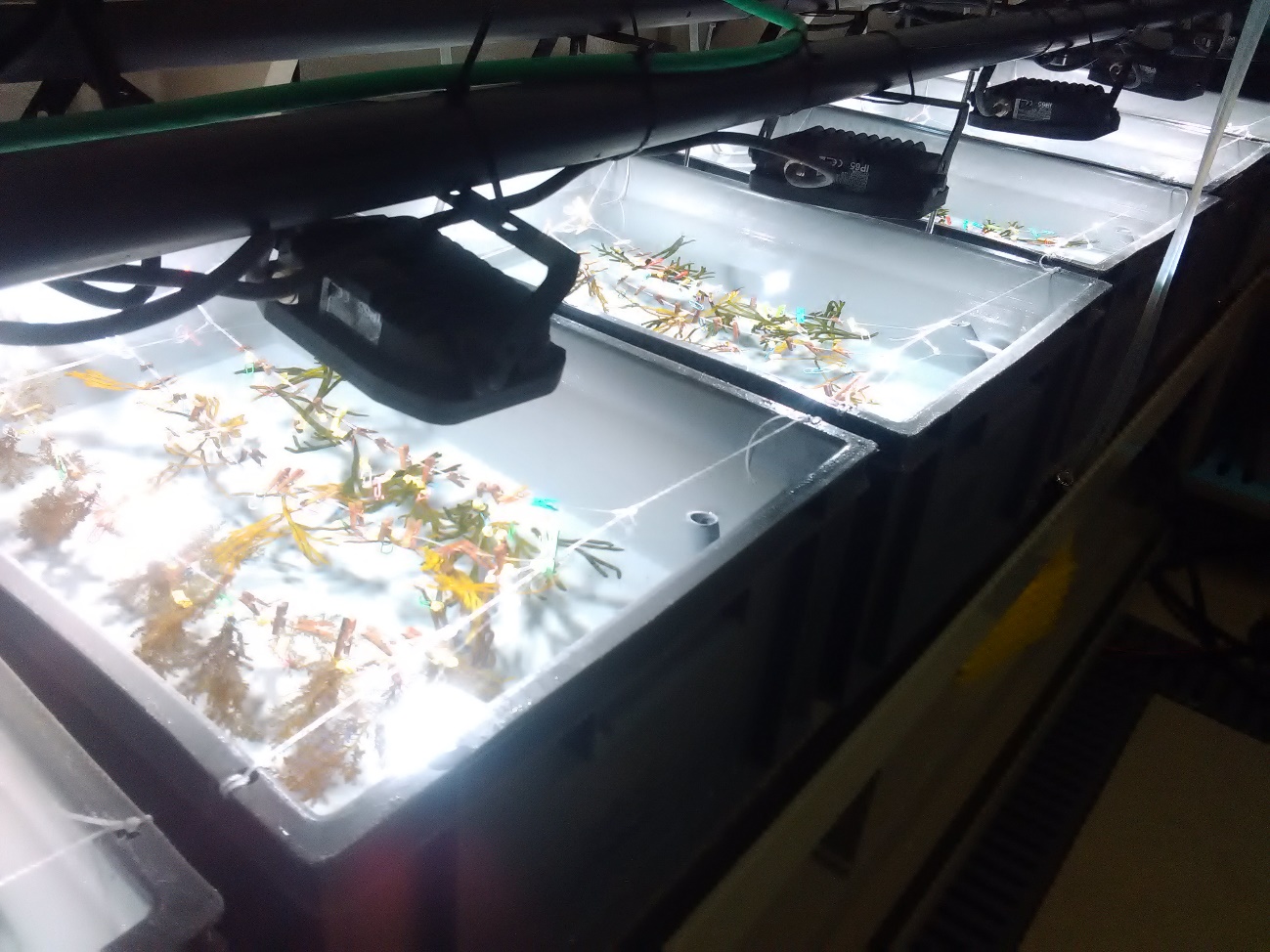


**SUPPLEMENTARY FIGURE S5**. Experimental set-up showing the distribution of macroalgal fronds in the tanks of seawater at different temperatures.

**
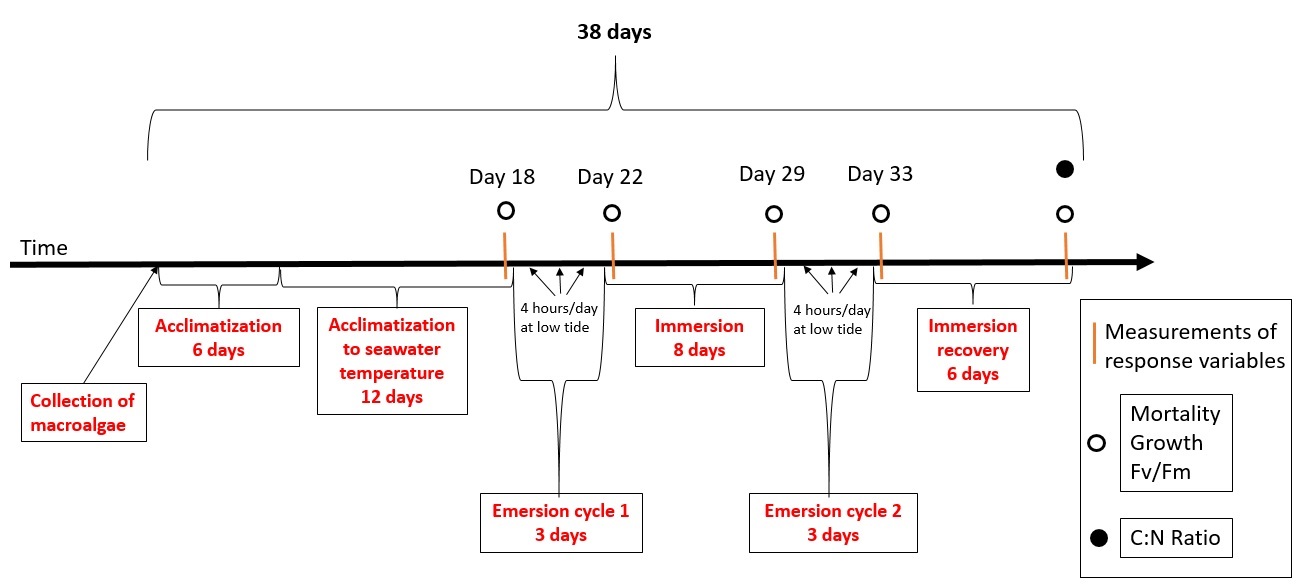
**

**SUPPLEMENTARY FIGURE S6.** Timing of the laboratory experiment.
